# Prevalence of multidrug resistant tuberculosis at tertiary care hospital

**DOI:** 10.1186/1471-2334-12-S1-P29

**Published:** 2012-05-04

**Authors:** Madhurima Pothula, P Sreenivasulu Reddy, P Vasundhara, Vedamurthy Reddy

**Affiliations:** 1 Narayana Medical College and General Hospital, Nellore, A.P, India

## Background

Tuberculosis continues to plague the world and remains the major global health problem. Simultaneously the incidence of drug resistant *Mycobacterium tuberculosis* strains is also increasing in almost all industrialized and developing countries.

## Methods

This prospective study was done at NMC, Nellore from July 2008-December 2009. Samples received at microbiology lab for acid fast staining were included in this study. Smears were stained by Ziehl-Neelsen’s technique. Samples were cultured on Lowenstein-Jensen media after processing by modified Petroff’s method and incubated according to CLSI guidelines. Identification of *Mycobaterium tuberculosis* was done based on morphology, nitrate reduction test and catalase test. Drug susceptibility for first line anti- tubercular drugs was performed by proportion ratio method.

## Results

A total of 2031 samples were included in this study. 120 samples were smear positive by acid fast staining, 110 were culture positive for *Myobacterium tuberculosis*. 16 (14.5%) samples were resistant to one or more antitubercular drugs. 10 (9.09%) samples showed monodrug resistance, Isoniazid (3.63%) followed by Rifampicin (2.72%) Ethambutol (1.81%) and Streptomycin (0.90%). Isoniazid and Ethambutol resistance in one sample (0.90%). Isoniazid and Rifampicin resistance in two samples (1.88%). Three samples were resistant to Isoniazid and Rifampicin along with other drugs (2.72%). HIV co-infection among MDR-TB was 2.7%.

## Conclusion

According to the present study prevalence of MDR-TB was 4.54%. Among the patients on treatment higher incidence of resistance was attributed to poor patient compliance in spite of effective DOTS programme.

